# ALA/LA ameliorates glucose toxicity on HK-2 cells by attenuating oxidative stress and apoptosis through the ROS/p38/TGF-β_1_ pathway

**DOI:** 10.1186/s12944-017-0611-6

**Published:** 2017-11-16

**Authors:** Mingxia Jiang, Haifen Zhang, Lijie Zhai, Bianliang Ye, Yin Cheng, Chengkai Zhai

**Affiliations:** 1 School of Public Health, Southeast University, Nanjing, 210009 China; 2 Jinling Hospital, Nanjing, 210002 China; 3 School of Tourism and Culinary Science, Yangzhou University, Yangzhou, 225127 China; 4 Department of Neurological Surgery, Northwestern University - Feinberg School of Medicine, Chicago, IL 60612 USA

**Keywords:** α-linolenic acid (ALA), linoleic acid (LA), HK-2 cells, glucose toxicity, oxidative stress, apoptosis, ROS/p38/TGF-β_1_ pathway

## Abstract

**Background:**

Growing evidence indicates that oxidative stress (OS) plays a pivotal role in Diabetic nephropathy (DN). In a previous study we demonstrated that ALA/LA protected HK-2 cells against high glucose-induced cytotoxicity. So we aimed to establish the glucose injury model of HK-2 cells and investigate the beneficial effects of ALA/LA on high glucose-induced excessive production of TGF-β1 and the possible mechanisms mediating the effects.

**Methods:**

The expression of OS markers in high glucose-induced HK-2 cells treated with ALA/LA., including the antioxidant enzymes and reactive oxygen species (ROS) production, as well as the apoptosis rate were assayed by ELISA and flow cytometry. The p38/transforming growth factor β_1_ (TGF-β_1_) signal pathway were measured by real-time RT-PCR and western blot.

**Results:**

The modeling condition of glucose toxicity on HK-2 cells was at the glucose concentration of 40.9 mM. ALA/LA can significantly increase the activities of antioxidant enzymes and decrease ROS production stimulated by high glucose. The study also found that ALA/LA caused a decrease in the apoptosis rate and TGF-β_1_ level of HK-2 cells under high glucose stress through the ROS/p38 pathway.

**Conclusions:**

ALA/LA exerts protective effects in vitro through inhibition of ROS generation, down regulation of the activation of the p38MAPK pathway and the expression of TGF-β_1_ in HK-2 cells.

## Background

Diabetic nephropathy (DN) is a major complication in patients with either type 1 or type 2 diabetes mellitus [1, 2]. It is also the leading cause of morbidity and mortality in patients with kidney disease worldwide. Tubular injury plays a critical role in DN progression, which correlates with renal functional deterioration, a primary change associated with the disease [3]. Accumulating data indicate that excessive oxidative stress (OS) and aberrant dynamics are the primary factors responsible for tubule damage in DN [4]. Hyperglycemia is the driving force for the development of DN; it increases the production of free radicals, such as reactive oxygen species (ROS), resulting in OS [5–7]. Furthermore, hyperglycemia-induced ROS activates p38 mitogen-activated protein kinase(MAPK), which induces phosphorylation of transcriptional factors, altered expression of genes, and production of fibronectin in mesangial cells, resulting in DN [8, 9]. Transforming growth factor β_1_ (TGF-β_1_) exerts biological activities through MAPK cascades in certain cell lines, especially through p38 MAPK in human mesangial cells and rat renal tubular cells [10, 11]. Moreover, several authors have reported that TGF-β_1_ was related to p38 MAPK in DN, suggesting a role of p38 MAPK as another important mediator of the TGF-β_1_ signal pathway [12]. Therefore, it is inferred that the kidney injury would be improved by decreasing the activation of the ROS/p38 MAPK/TGF-β_1_ signal pathway.

Human kidney (HK-2) cells, a proximal tubular epithelial cell line derived from normal adult human kidney cells immortalized by transduction with human papillomavirus (HPV 16) DNA fragment [13].It has been found that these cells retain most of functional characteristics, i.e. OS, enzymes, cytochromes, consistent with in vivo system and human proximal tubular cells [14, 1]. So that HK-2 cells were used in the experiments on nephrology and nephrotoxicity.

Linoleic acid (LA; 18:2, n-6) and α-linolenic acid (ALA; 18:3, n-3) are essential fatty acids for humans and many animals and are important constituents of membranes. Recent studies have shown that dietary supplementation with n-3 polyunsaturated fatty acids (PUFAs) retards the progression of renal diseases in humans and animals [15, 16]. Adriano et al. [17] reported that dietary n-3 PUFAs have beneficial effects against OS in DM by decreasing ROS production and increasing the activities of superoxide dismutase (SOD), catalase (CAT), and glutathione peroxidase (GSH-PX). Our previous research also indicated that ALA was a protectant to prevent pig kidney proximal tubular cells (LLC-PK1) from high glucose-induced injuries by suppressing ROS generation and apoptosis [18]. In addition, ALA ameliorates proteinuria by down-regulating the expression of TGF-β_1_ and fibronectin proteins, and these favorable effects are related to the inhibition of phosphorylating activation of the p38 MAPK pathway in the renal cortex of OLETF rats [12]. However, little is known about the effect of both n-3 and n-6 PUFAs on HK-2 cells induced by high glucose. Our research investigated the relationship of ALA/LA and OS and apoptosis in the condition of high glucose and explored the possible mechanisms mediating the effects of ALA/LA.

## Methods

### HK-2 cell high glucose injury model

HK-2 cells were purchased from American Type Culture Collection (ATCC). HK-2 cells were grown in Dulbecco modified Eagle medium (DMEM):F12(Gibco, NY, USA) with 15 mM *N*-2-hydroxyethylpiperazine -*N*-2-ethanesulfonic acid (HEPES) (Sigma-Aldrich, MO, USA), L-glutamine (Sigma-Aldrich, MO, USA), and pyridoxine HCl (Gibco, NY, USA), supplemented with 10% (*v*/v) fetal calf serum (FCS, Gibco, NY, USA) at 37 °C in 95% humidified air and 5% CO_2_. All experiments were performed under serum-free conditions in which the cells remained viable in a nonproliferating state. Seeded cells (3 × 10^5^ cells/mL) were treated with ALA, LA, timnodonic acid (EPA) (Nu-Chek, MN, USA), and ALA/LA (10–200 μM) for 48 h. For inhibition assays, the cells were first treated with inhibitors for 1 h before stimulation. After all treatments, the medium was collected and centrifuged. The cell-free supernatant was then stored at 80 °C for future assays. The cells remaining on the plates were rinsed with phosphate-buffered saline (PBS) and harvested.

### Experimental design

**Fig. 1:**
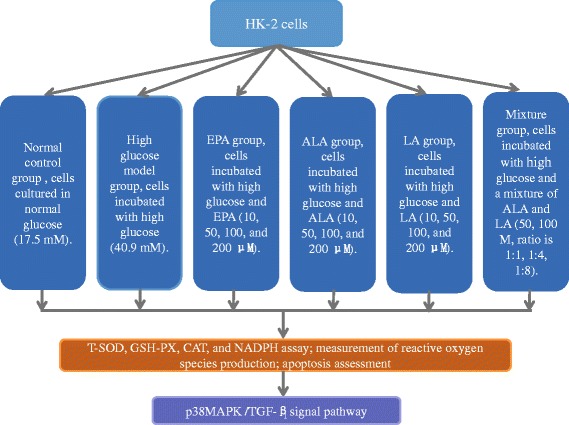
Experimental groups and treatments on HK-2 cells

In the experiments, HK-2 cells were divided into six groups: normal control group, high glucose model group, EPA treatment group, ALA treatment group, LA treatment group and mixture of ALA and LA treatment group, as shown in Fig. 1. Because of the excellent antioxidant ability of EPA [19–21], the EPA treatment group acts as a positive control group. The cells were harvested at the indicated time to evaluate cell viability, apoptosis and ROS.

### T-SOD, GSH-PX, CAT, and NADPH assay

The activities of superoxide dismutase (SOD), catalase (CAT), and glutathione peroxidase (GSH-PX) were measured using enzyme-linked immunosorbent assay (ELISA) kits (Nanjing Keygen Biotech Co., Nanjing, China), according to the manufacturer’s instructions. All samples were analyzed in triplicate, and a separate standard curve was run for each ELISA plate. All samples were measured at 450 nm using a microplate reader (Bio-Rad, CA, USA).

### Measurement of reactive oxygen species (ROS)production

The ROS production was detected by a commercially available kit (Beyotime Institute of Biotechnology, Haimen, China). HK-2 cells were seeded in 6-well microplates at 2 × 10^5^ cells/well for 12 h, and then the cells were incubated with different treatments for 48 h. After incubation, cells were collected, counted and washed with PBS. Then cells (1 × 10^7^/mL) were added into 10 μM DCFH-DA in a volume of 1 mL/group. Cells were stained at 37.C for 20 min, and washed with PBSto remove the free DCFH-DA molecules. ROS generation was assessed using flow cytometry (Becton, Dickinson and Company, NJ, USA).

### Apoptosis assessment

HK-2 cells (10^6^) were washed twice with PBS and double stained with fluorescein isothiocyanate (FITC)-conjugated Annexin V (Invitrogen, CA. USA) and PI (Invitrogen, CA. USA) according to the manufacturer’s instructions. The samples were analyzed immediately on the flow-cytometer (Becton, Dickinson and Company, NJ,USA). Cells negative for both Annexin V-FITC and PI were considered to be living cells; those positive for Annexin V-FITC and negative for PI were considered to be early apoptotic cells; and those positive for both dyes were considered to be late apoptotic or necrotic cells.

Data analysis was performed using the software WinMDI 2.9 (Invitrogen, CA, USA).

### Measurement of real-time reverse transcription-polymerase chain reaction (RT-PCR) for TGF-β_1_ and p38mRNA

Total RNA was extracted using the method of Chomczynski and Sacchi [22], and the cDNA was synthesized by RT-PCR using 2 μg of total RNA with oligo-dT primers (Promega, WI, USA). Real-time RT-PCR was performed using a Light Cycler (Roche, Basel. Switzerland). The forward and reverse primers for TGF-β_1_, p38, and GADPH (Sangon, Shanghai, China) are shown in Table 1. The amplification was performed with the following time course: starting at 95 °C for 10 min, followed by denaturation at 95 °C for 15 s, then annealing at 60 °C for 60 s, and extension for 40 cycles as the last step. The detection of fluorescent products was carried out at the end of the 60 °C extension period. Each sample was tested twice. To confirm the amplification specificity, the PCR products were subjected to melting curve analysis and subsequent 1.5% agarose gel electrophoresis. For each sample, the cycle threshold (ΔCt = C _TGF-β1/p38_ - Ct _GADPH_) was calculated according to the published method [23]. The relative changes in TGF-β_1_/GADPH and p38/GADPH mRNA ratio between the control and experimental conditions were determined by the formula = 2^-ΔΔCt^, where ΔΔCt is the difference in ΔCt between the control and experimental conditions.

**Table 1:** Sequences of primers for realtime RT-PCR

Genes	Sequences	Length
GAPDH	F 5′ AATCCCATCACCATCTTC 3’	218 bps
	R 5′ AGGCTGTTGTCATACTTC 3’	
TGF-β_1_	F 5′ GACTACTACGCCAAGGAGGTC 3’	149 bps
	R 5′ GAGAGCAACACGGGTTCAG 3’	
P38	F 5′ ACCTACAGAGAACTGCGGTTAC 3’	124 bps
	R 5’TGAGATGGGTCACCAGATACAC 3’	

### Western blot analysis

Cells (2 × 10^5^) were added to 10-mm dishes pre-incubated with ALA/LA (100 μM) for 12 h, and then incubated with high glucose (40.9 mM) and ALA/LA (100 μM) for 48 h. The cell lysates were prepared with ice-cold lysis buffer containing 20 mM Tris-HCl (pH 8.0); 1 mM sodium orthovanadate; 10% glycerol; 1 mM phenylmethylsulfonyl fluoride; 2 mM EDTA; 1% Triton X-100; 50 mM β-glycerolphosphate; and 10 mg/mL each of aprotinin, leupeptin, and pepstatin. A total of 25 μg proteins were determined by the BCA Protein Assay Kit (Beyotime Institute of Biotechnology, Haimen, China), and they were resolved by SDS-PAGE using 10% gel and transferred to polyvinylidene difluoride (PVDF) (Millipore Co., MA, USA) blocked with Tris-buffered saline (TBS) containing 5% (*w*/*v*) nonfat dry milk and 0.1% (*v*/v) Tween-20 for more than 2 h. The membrane was then probed with primary antibodies against GADPH (1:1500), TGF-β_1_ (1:800), p38 (1:1000), and p-p38 (1:800) at 4 °C overnight. After washing, the membrane was incubated with HRP-conjugated goat anti-rabbit IgG (1:1000) or HRP-conjugated goat anti-mouse IgG (1:1000) at room temperature for 1 h. Immunodetection was performed with an enhanced chemiluminescence (ECL) detection kit (Cell Signaling Technology, MA, USA) and Konica X-ray film system (Konica, Tokyo, Japan).

### Statistical analysis

Results are shown as mean ± standard error of three individual assays. Statistical analyses were performed following one-way analysis of variance (ANOVA) and Tukey’s test using SPSS 18.0 (SPSS, Chicago, USA). Values were considered statistically significant when *P* was less than 0.05 or 0.01.

## Results

### High glucose-induced HK-2 cells injury

The viability of the cells was determined by the MTT assay. Our previous study showed a significant reduction of cell survival after 48 h of treatment with high glucose in concentrations of 25.3, 33.1, 40.9, 48.8, 56.6, 64.6, 72.2,80, 87.8, and 95.3 mM (*P* < 0.05 or *P* < 0.01), and the dose-effect relationship was obvious (*r* = − 0.91,*P* < 0.05). The viability of HK-2 cells was reduced to 43.4% with the treatment of high glucose at a dose of 56.6 mM for 48 h [24].

### ALA/LA elevated the level of SOD, GSH-PX, and CAT and reduced the level of NADPH-PX in HK-2 cells induced with high glucose

As shown in Table 2, there was a significant reduction in the level of SOD, GSH-PX, CAT, and a significant increase in NADPH-PX in the high glucose model group, as compared to those in the normal control group (*P* < 0.05). On the contrary, after intervention, the levels of SOD, GSH-PX and CAT in the ALA intervention group, LA intervention group, EPA intervention group, and the mixture of ALA and LA intervention group were increased, while the levels of NADPH-PX in those groups were declined.

**Table 2:** Effect of ALA/LA on SOD, GSH-PX, CAT and NADPH-PX level of HK-2 cell induced with high glucose ($\overline{x}$ ±s)

group	dose (μM)	SOD (mM)	GSH-PX (mM)	CAT (mM)	NADPH-PX(mM)
N	0	185.66 ± 15.90^*^	207.40 ± 18.29^*^	6.61 ± 0.60^*^	37.30 ± 3.66^*^
G	0	83.64 ± 8.50^#^	73.86 ± 18.39^#^	3.59 ± 0.54^#^	86.52 ± 3.20^#^
A_1_	10	286.00 ± 19.53^**^	87.29 ± 10.23	6.39 ± 0.73^*^	63.85 ± 1.97^*^
A_2_	50	304.50 ± 8.46^**^	107.43 ± 22.04	6.21 ± 0.86^*^	56.13 ± 3.79^*^
A_3_	100	252.24 ± 16.73^*^	161.79 ± 30.77^*^	3.28 ± 0.36	54.08 ± 4.04^*^
A_4_	200	229.97 ± 44.72^*^	59.42 ± 12.98	2.25 ± 0.40	68.01 ± 3.11^*^
L_1_	10	215.54 ± 11.98^*^	73.99 ± 18.71	6.41 ± 0.49^*^	46.62 ± 0.98^*^
L_2_	50	189.76 ± 26.83^*^	185.27 ± 21.02^*^	4.17 ± 0.53	43.92 ± 2.82^*^
L_3_	100	155.28 ± 32.11	209.33 ± 23.30^*^	2.27 ± 0.72	48.01 ± 2.39^*^
L_4_	200	155.59 ± 13.68	107.46 ± 25.81	4.32 ± 0.43	54.28 ± 4.30^*^
E_1_	10	262.93 ± 34.99^*^	96.16 ± 16.33	3.57 ± 0.51	80.56 ± 3.3
E_2_	50	252.33 ± 6.56^*^	50.17 ± 11.58	3.68 ± 0.62	71.22 ± 2.82^*^
E_3_	100	261.65 ± 6.14^*^	107.61 ± 22.73	7.61 ± 1.93^*^	76.17 ± 2.80^*^
E_4_	200	247.74 ± 11.99^*^	53.72 ± 12.08	2.13 ± 0.38	82.66 ± 3.22
M_1_	50 (1:1)	249.80 ± 13.62^*^	107.61 ± 22.73	12.66 ± 2.34^*^	82.91 ± 3.95
M_2_	50 (1:4)	256.60 ± 21.92^*^	201.73 ± 24.21	5.76 ± 0.77	74.51 ± 2.32^*^
M_3_	50 (1:8)	246.53 ± 45.78^*^	59.71 ± 12.28	3.65 ± 0.67	80.77 ± 2.24
M_4_	100 (1:1)	273.29 ± 15.59^*^	178.99 ± 40.81^*^	6.30 ± 0.96^*^	63.38 ± 4.25^*^
M_5_	100 (1:4)	221.32 ± 26.12^*^	230.95 ± 44.38^*^	3.32 ± 1.47	42.78 ± 1.27^*^
M_6_	100 (1:8)	240.02 ± 41.92^*^	135.41 ± 27.25	3.13 ± 0.81	63.39 ± 2.47^*^

Data are presented as mean ± SEM. ^#^ *P* < 0.05 vs. control group. ^*^ *P* < 0.05, ^**^ *P* < 0.01 vs. model group. N: normal control group; G: high glucose model group; A_1_-A_4_: ALA group at dose of 10, 50, 100, 200 μM; L_1_-L_4_:LA group at dose of 10, 50, 100, 200 μM; E_1_-E_4_ EPA group at dose of 10, 50, 100, 200 μM, M_1_-M_3_: mixture of ALA and LA group at dose of 50 μM with ALA/LA ratio of 1:1, 1:4, 1:8; M_4_-M_6_: mixture of ALA and LA group at dose of 100 μM with ALA/LA ratio of 1:1, 1:4, 1:8

### ALA/LA decreased ROS level in HK-2 cells induced with high glucose

**Fig. 2:**
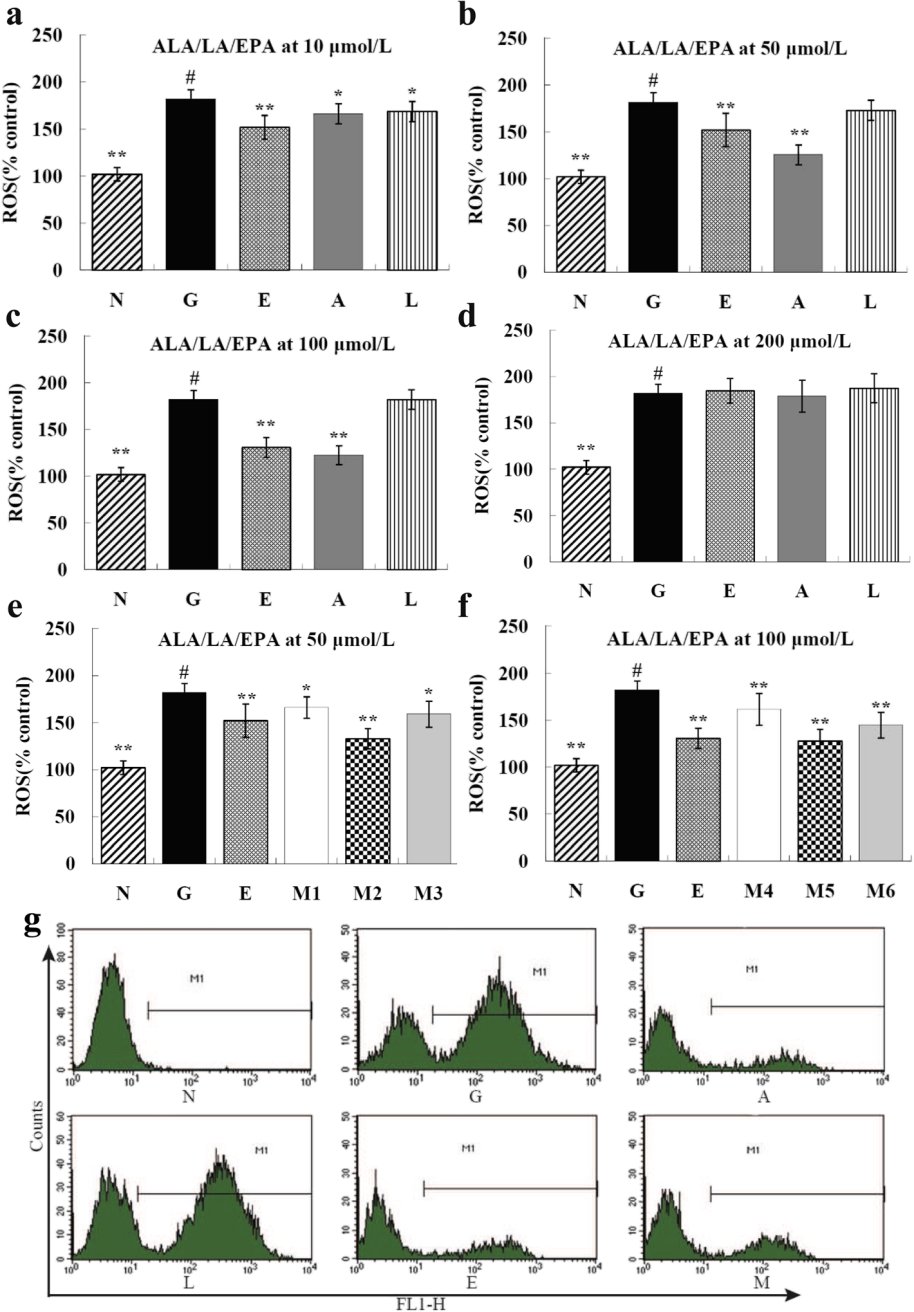
Effects of ALA/LA on ROS level of HK-2 cells induced with high glucose ($\overline{x}$ ±s). ROS generation of HK-2 cell treated with ALA/LA was evaluated by flow cytometery. Data are presented as mean ± SD. ^#^ *P* < 0.05 vs. control group. ^*^ *P* < 0.05, ^**^ *P* < 0.01 vs. model group. N: normal control group; G: high glucose model group; A_1_-A_4_: ALA group at dose of 10, 50, 100, 200 μM; L_1_-L_4_:LA group at dose of 10, 50, 100, 200 μM; E_1_-E_4_ EPA group at dose of 10, 50, 100, 200 μM, M_1_-M_3_: mixture of ALA and LA group at dose of 50 μM with ALA/LA ratio of 1:1, 1:4, 1:8; M_4_-M_6_: mixture of ALA and LA group at dose of 100 μM with ALA/LA ratio of 1:1, 1:4, 1:8. **a** to **f** Effects of ALA, LA, EPA, ALA and LA with a different concentration on ROS generation of HK-2 cells induced with high glucose. **g** ROS level of HK-2 cell treated with ALA/LA (100 μM)

Compared to the normal control group, high glucose-induced intracellular ROS generation of HK-2 cells in the high glucose model group was 1.8 times higher (*P* < 0.01) (Fig. 2a-f). The 48 h intervention of ALA, LA, EPA, and ALA/LA caused a significant reduction in ROS generation (*P* < 0.01 or *P* < 0.05) in high glucose-induced HK-2 cells, when ALA and EPA were at doses of 10 μM, 50 μM, and 100 μM; LA was at the dose of 10 μM; ALA/LA was at the dose of 50 μM and 100 μM with ALA/LA ratios of 1:1, 1:4, and 1:8 (Fig. 2a-f). It should be noted that ALA at the dose of 100 μM showed the best effect in all groups. The production of ROS was 2.6 times decreased than the amount in the high glucose model group (*P* < 0.01). Results are shown in Fig. 2c and Fig. 2g.

### ALA/LA inhibited apoptosis of HK-2 cells induced with high glucose

**Fig. 3:**
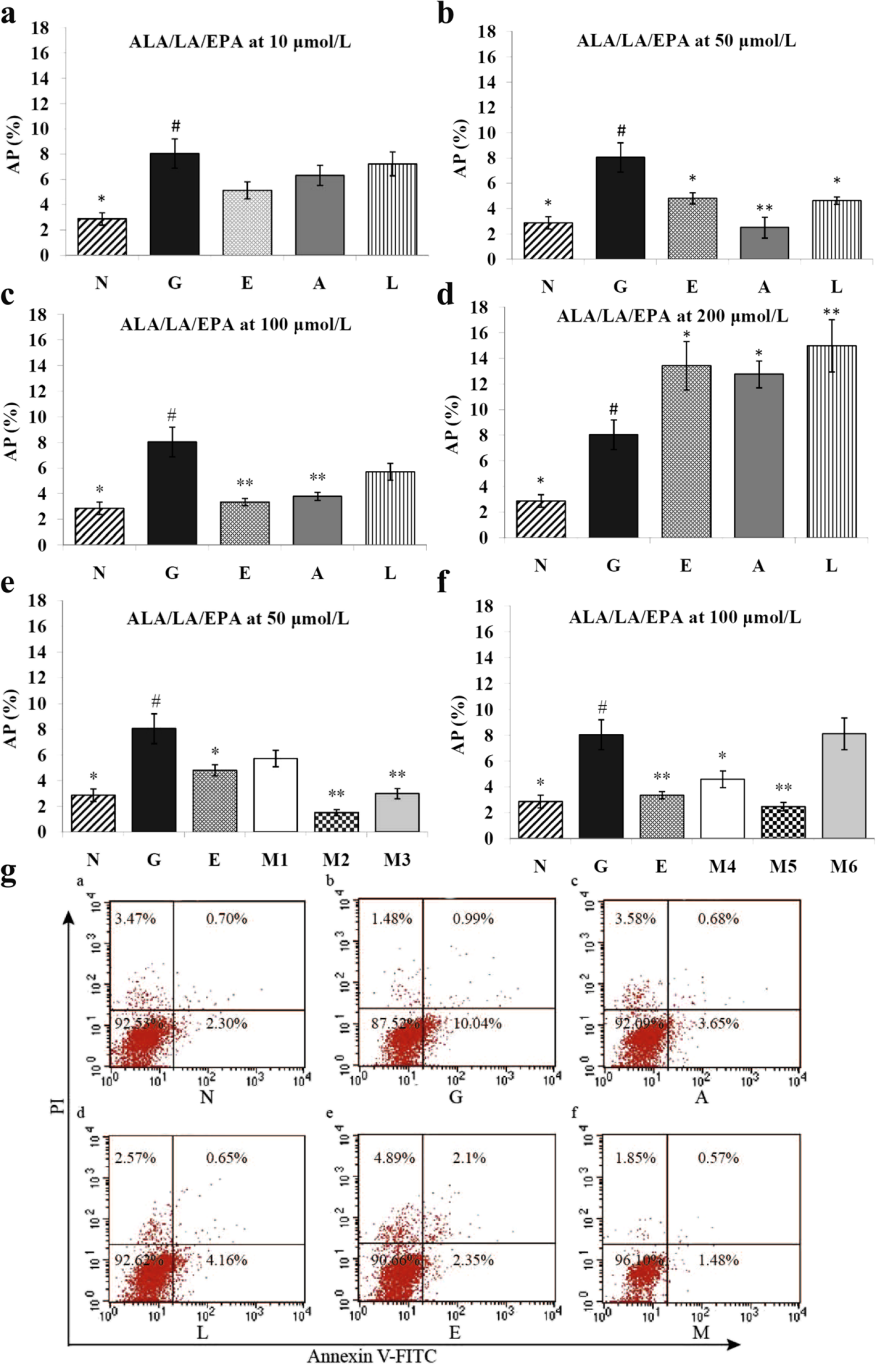
Effects of ALA/LA on apoptosis of HK-2 cells induced with high glucose($\overline{x}$ ±s). Apoptosis percents of HK-2 cell treated with ALA/LA were evaluated by flow cytometery. N: normal control group; G: high glucose model group; A, L, E: ALA/LA/EPA intervention group at dose of 50 μM; M: mixture of ALA and LA intervention group at dose of 100 μM with ALA/LA ratio of 1:4. **a** to **f** Effects of ALA, LA, EPA, ALA and LA with a different concentration on apoptosis of HK-2 cells induced with high glucose. **g** Apoptosis percents of HK-2 cell treated with ALA/LA (100 μM)

To determine whether apoptosis was associated with OS, we also measured the apoptosis rate after intervention of free fatty acid. As shown in Fig. 3, the apoptosis rate of HK-2 cells in the high glucose model group was significantly higher than that in the normal control group (*P* < 0.05). Moreover, we found that the apoptosis rates were significantly inhibited after 48 h incubation with 50 μm and 100 μM ALA, 50 μM of LA, 50 μM of ALA/LA with ratios of 1:4 and 1:8, and 100 μM ALA/LA with ratios of 1:1 and 1:4 (*P* < 0.05 or *P* < 0.01). The apoptosis rate was lowest in the intervention of ALA/LA at a dose of 50 μM with a ratio of 1:4 (*P* < 0.01) (Fig. 3b and Fig. 3g). Interestingly, there was a significant increase in apoptosis rate of HK-2 cells with 200 μM ALA, LA, and EPA (*P* < 0.01) (Fig. 3d).

### ALA/LA down regulated mRNA expression of the p38/TGF-β_1_ signal pathway in high glucose-induced HK-2 cells

**Fig. 4:**
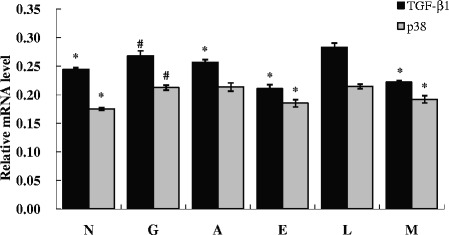
Effects of ALA/LA on TGF-β_1,_ p38 mRNA levels of HK-2 cells induced by high glucose (100 μM). Relative mRNA abundances of TGF-β_1_ and p38 in HK-2 cell induced by high glucose were measured. The steady-state mRNA concentrations of TGF-β_1_ and p38 were quantified with real-time PCR and 2^-ΔΔCt^ values were calculated to obtain fold expression levels. GAPDH mRNA levels were similarly measured and served as a reference control for mRNA quality and quantity. Bars represent the means ± SD of relative levels. *# Means not sharing a common symbol are significantly (*P* < 0.05) different from each other. N: normal control group; G: high glucose model group; A, L, E: ALA/LA/EPA intervention group at dose of 100 μM; M: mixture of ALA and LA intervention group at dose of 100 μM with ALA/LA ratio of 1:4

As demonstrated in our study, ALA/LA treatment caused a change in ROS and apoptosis of high glucose-induced HK-2 cells. Further, we focused on the p38/TGF-β_1_ signal pathway and found that at the dose of 50 μM, ALA, EPA, and ALA/LA decreased the mRNA expression of TGF-β_1._ EPA and ALA/LA at a dose of 50 μM also inhibited the mRNA expression of p38 (Fig. 4).

### ALA/LA inhibits the phosphorylation of p38 and TGF-β_1_ expression of high glucose-induced HK-2 cells

**Fig. 5:**
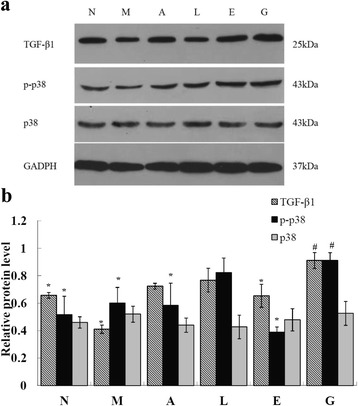
TGF-β_1_, p-p38 and p38 expression in high glucose induced HK-2cells with ALA/LA intervention (100 μM). **a** Protein expression of TGF-β_1_ and p38 in high glucose induced HK-2 cells with ALA/LA intervention by western blot. **b** The intensity of the bands was quantified by densitometry analysis and normalized with corresponding GADPH. Values are expressed as the mean ± SD Bars without a common superscript symbol indicate significant differences among groups at *P* < 0.05. N: normal control group; G: high glucose model group; A, L, E: ALA/LA/EPA intervention group at dose of 100 μM; M: mixture of ALA and LA intervention group at dose of 100 μM with ALA/LA ratio of 1:4

**Fig. 6:**
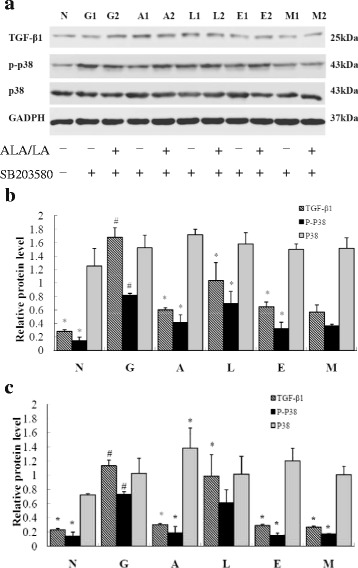
TGF-β_1_, p-p38 and p38 expression, assessed by western blot, in high glucose induced HK-2cells with ALA/LA and/or SB203580 intervention (100 μM). **a** Protein expression of TGF-β_1_ and p38 in high glucose induced HK-2cells with ALA/LA and/or SB203580 by Western blot. **b** The effects of SB203580 on TGF-β_1_, p-p38 and p38 expression in high glucose induced HK-2cells. **c** The effects of ALA/LA and SB203580 (the inhibitor of p38) on TGF-β_1_, p-p38 and p38 expression in high glucose induced HK-2cells.The intensity of the bands was quantified by densitometry analysis and normalized with corresponding GADPH. Values are expressed as the mean ± SD. Bars without a common superscript symbol indicate significant differences among groups at *P* < 0.05. N: normal control group; G: high glucose model group; A, L, E: ALA/LA/EPA intervention group at dose of 100 μM; M: mixture of ALA and LA intervention group at dose of 100 μM with ALA/LA ratio of 1:4

The relationship between TGF-β_1_ and end stage renal disease has been proved [10]. To further investigate the mechanisms underlying the high glucose-induced damage effects, we measured the TGF-β_1_ expression in HK-2 cells by western blot analysis (Fig. 5 and Fig. 6). There was a significant difference in TGF-β_1_ expression between the normal control group and the high glucose model group (*P* < 0.05), but after intervention of 100 μM EPA and 100 μM ALA/LA with a ratio of 1:4 for 48 h, the TGF-β_1_ expression significantly decreased in HK-2 cells (*P* < 0.05).

Previous studies by other groups demonstrated that TGF-β_1_ is activated mainly through the MAPK signal pathway [25, 26]. As shown in Fig. 5 and Fig. 6, in the high glucose model group, the expression of p38, a critical subunit of MAPK, was markedly increased compared with in the normal control group in HK-2 cells (*P* < 0.05), and was partly restored by ALA/LA intervention (*P* < 0.05). There was no significant difference in p38 expression among the different groups, whereas 100 μM ALA and EPA and 100 μM ALA/LA with a ratio of 1:4 intervention for 48 h significantly reduced the phosphorylation of p38 in HK-2 cells (*P* < 0.05). These results suggest that a decrease in p38 expression may play a causative role in the TGF-β_1_ activation in HK-2 cells. These results indicate that ALA intake may exert its kidney protection, including anti-oxidative stress effects, through the activation of TGF-β_1_ in a p38-dependent manner in high glucose-induced HK-2 cells.

## Discussion

In this study, we used a high glucose-induced HK-2 cell line as a model to investigate the effects of ALA/LA on DN. The results indicate that high glucose induced glucose toxicity on HK-2 cells. It showed the same effect on LLC-PK_1_ cells [18].Furthermore, our results showed that the viability of HK-2 cells was lowest with the treatment of high glucose at a dose of 56.6 mM for 48 h,shown in Fig. 2. However, with reference to other studies [27], and considering that a too low survival viability of HK-2 cells may interfere with the study’s results, a better condition to establish a glucose toxicity model of HK-2 cells was treatment with high glucose at dose of 40.9 mM for 48 h.

Increased ROS levels under high-glucose conditions induce OS, which can result in the development of a variety of biochemical and physiological lesions. Such cellular damage frequently impairs the metabolic function and results in cell death [13]. On the basis of these results of glucose toxicity on HK-2 cells [24] and the previous research [18], the exposure of HK-2 cells to high levels of glucose resulted in significant reduction in the cell viability. However, ALA/LA treatment was also shown to inhibit cell death, thereby suggesting that ALA/LA protects HK-2 cells against high glucose-induced cytotoxicity. Interestingly, in most cases ALA, LA and ALA/LA have these good function in low or medium concentration. Because polyunsaturated fatty acid may occur peroxidation at high dose, and it will show harmful effect in this case [28].

To combat and prevent OS, cells contain sophisticated intracellular antioxidant defense systems, including an enzymatic antioxidant system and a non-enzymatic antioxidant system. The former mainly comprises SOD, CAT, GSH-PX, and glutathione reductase (GR). The latter includes the antioxidants glutathione (GSH) as well as ascorbic acid, tocopherol, and carotenes [29]. However, there is an imbalance between the oxidant and anti-oxidant mediators prior to the development of renal lesions, and the oxidation level increases as the disease progresses, such as in DN [30]. Several studies reported that high-glucose conditions induced ROS production over antioxidant defense systems damage [31–35], and our study showed the same results (Table 2 and Fig. 3). We have also found that ALA/LA can protect HK-2 cells from oxidative stress induced by high glucose not only by scavenging intracellular ROS, but also by enhancing endogenous antioxidant defense systems including the antioxidant enzyme defense system and the GSH system. Comparable results have been reported by Shen et al. [36], who found that an increased level of high glucose caused slightly increased ROS generation, which correlated with a decrease in SOD activity. ALA suppressed ROS generation to a significant degree in a dose-dependent fashion and increased SOD activity significantly. In our previous work, the antioxidative capacity of ALA/LA was determined on the basis of the intracellular ROS clearance capacity in LLC-PK_1_ cells that had been oxidized by high glucose [18].

The antioxidant function of ALA/LA is thought to be related to the regulation of expression of the genes involved in ROS metabolism and/or ROS-dependent signaling pathways, such as the MAPK signaling pathway [37–40]. This could suggest that the ALA/LA activates the related intracellular antioxidant signaling pathways and gene expression only when oxidative stress occurs [41]. Additional studies are needed to test this hypothesis and to explain the precise mechanism of action by which antioxidant peptides in cells regulate intracellular ROS scavenging activity and antioxidant defense systems.

Previous studies suggested that ROS are generated as an early signal in human tubular cells, which subsequently develop apoptotic changes under high-glucose media, implicating ROS as potential mediators of glucose-induced apoptosis [42, 43]. In addition, based on recent reports regarding the relationship between ROS and MAPKs in apoptosis, it has been well established that p38 activation contributes to a positive feedback loop, such as the ROS/p38 MAPK cascade for cell apoptosis [44]. We investigated the apoptotic effect of ALA/LA and the underlying molecular mechanism for glucose toxicity on HK-2 cells. The data showed that the production of both ROS and p38 is involved in high glucose-induced apoptosis and that these effects were recovered by ALA/LA treatment, which was a ROS scavenger (Fig. 3 and Fig. 4). Furthermore, the results confirmed that ALA/LA treatment reduced high glucose-induced activation of p38, and SB203580 (p38 inhibitor) (Fig. 5 and Fig. 6) partially reduced ROS production. Taken together, these results demonstrate that ALAL/LA decreased the apoptosis induced by high glucose through the ROS/p38 signal pathway.

Diabetic nephropathy is characterized histologically by glomerular basement membrane thickening and mesangial matrix expansion, resulting in glomerulosclerosis [45]. TGF-β_1_ is known as a key mediator of the sclerosing process in diseased glomeruli and is related to glomerulosclerosis and interstitial fibrosis in various renal diseases [46, 47]. Several authors have described that elevated TGF-β_1_ production was induced by exposure to high glucose in the diabetic kidney [48, 49]. Our data showed that the mRNA and protein level of TGF-β_1_significantly increased in HK-2 cells in the condition of glucose toxicity compared with the control group. However, ALA/LA attenuated the increased expression of TGF-β_1_ in HK-2 cells in the condition of glucose toxicity. Thus, our aim was also to investigate the beneficial effects of ALA/LA on high glucose-induced excessive production of TGF-β_1_ and the possible mechanisms mediating the effects. It is well known that ROS and MAPK are closely related to TGF-β_1_. First, it was presence of excessive ROS in the kidney that can directly promote the activation of multiple OS-related signaling pathways, including the p38MAPK pathways, as well as the overexpression of downstream various fibrogenic cytokines containing TGF-β_1_. Consequently, it is possible to improve renal fibrosis and delay the progress of DN by reducing OS and regulating OS-related signaling pathway activities in the kidney [50, 51]. Our study show that high glucose triggered ROS overproduction and high expression of p38 and TGF-β_1_ in HK-2 cells. Moreover, after intervention with 100 μM ALA and 100 μM ALA/LA with a ratio of 1:4, ROS generation, the mRNA level of TGF-β_1_ and p38 MAPK, and the protein level of TGF-β_1_ and phosphorylated p38 MAPK were decreased. In conclusion, high glucose induces OS and apoptosis of HK-2 cells. The therapeutic actions of anti-oxidants in vitro are directly related to controlling the activities of intracellular signaling pathways associated with OS, in which the p38MAPK pathways occupy the key positions in DN at the early stage. The potential mechanisms by which ALA/LA exerts protective effects against in glucose toxicity in HK-2 cells in vitro are through inhibition of ROS generation, down regulating the activation of the p38MAPK pathway, and the expression of TGF-β_1_. Thus, our study provided new insights into the role of ALA/LA in high glucose-induced OS in HK-2 cells through the ROS/p38MAPK /TGF-β_1_ signal pathway.

## Conclusions

The results of the present study concluded that high glucose induced OS and glucose toxicity of HK-2 cells.. While ALA/LA exerts protective effects in vitro through inhibition of ROS generation, down regulation of the activation of the p38MAPK pathway and the expression of TGF-β_1_ in HK-2 cells.

## Abbreviations

ALA: α-linolenic acid
CAT: Catalase
DN: Diabetic nephropathy
EPA: Timnodonic acid
GSH-PX: Glutathione peroxidase
HK-2: Human tubular epithelial cell
LA: Linoleic acid
MAPKs: Mitogen -activated protein kinase
NADPH-PX: Nicotinamide adenine dinucleotide phosphate oxidase
OS: Oxidative Stress
ROS: Reactive oxygen species
SOD: Superoxie pismutese
TGF-β_1_: Transforming growth factor-β_1_
